# Modelling and predicting fat deposition rates in various South African sheep crosses using ultrasound technology

**DOI:** 10.1007/s11250-023-03732-y

**Published:** 2023-09-26

**Authors:** P. G. Theron, T. S. Brand, S. W. P. Cloete, J. H. C. van Zyl

**Affiliations:** 1 Department of Animal Sciences, Stellenbosch University, Private Bag X1, Matieland, 7602 South Africa; 2 Directorate: Animal Sciences, Department of Agriculture, Western Cape Government, Private Bag X1, Elsenburg, 7607 South Africa

**Keywords:** Carcass classification, Crossbreeding, Predictive models, Slaughter readiness

## Abstract

Producers require an accurate predictive tool that can determine the optimal point of slaughter based on fat depth. The modelling of fat deposition with a simple mathematical model could supply in this need. Dohne Merino and Merino ewes were crossed with Dorper, Dormer and Ile de France rams or rams of their own breeds to create two purebred (Dohne Merino and Merino) and six crossbred groups (Dohne x Dorper, Dohne x Dormer, Dohne x Ile de France, Merino x Dorper, Merino x Dormer and Merino x Ile de France) of offspring. Fat deposition of four lambs of each sex per genotypic group was monitored from 80 to 360 days using ultrasound, and the data subsequently fitted to various equations and evaluated for goodness of fit. A linear fitting of fat depth to age (*R*^2^ > 0.77) and live weight (*R*^2^ > 0.56) were deemed to provide the best fit. The slope parameters of the equations indicated that ewes deposited fat faster than rams and that Dorper crosses had the highest fat deposition rate. An attempt was also made to model loin muscle growth, but the model fit was judged to be unsatisfactory. The predictive models developed here are deemed suitable for inclusion in feedlot management systems to aid in the production of optimally classified lamb carcasses.

## Introduction

In South Africa, carcass classification is regulated by government legislation, and carcasses are classified according to both age and fatness (Government Notice R. 863, 2006). There are four age (A, AB, B and C) and six fatness (0–6) classes, with the most valuable being A2/A3 carcasses. These are lamb (A) carcasses with a backfat depth of 1–4 mm (2) or 4–7 mm (3). Consumers prefer meat from these carcasses as enough fat is present to ensure juicy and tender cuts, but the carcasses are not over-fat (Cloete et al., 2007; Webb and O’Neill, 2008).

Given that breeds have certain genetic limitations regarding growth and production traits (Brand and Franck, 2000), feedlotters may opt to use crossbred animals and so combine desirable traits from two parental breeds in order to increase their production output. Crossbred lambs are more likely to ensure economic success in a feedlot (Duddy et al., 2016), while it is commonly accepted that crossbreeding can improve lamb production (Sidwell and Miller, 1962; Fahmy et al., 1972; Cloete et al., 2007, 2008). Malhado et al. (2009) believed that crossbreeding could be used to meet specific market needs. However, regardless of the production potential of a feedlot lamb, the producer will lose potential income if it is slaughtered at non-optimal fat cover.

In most feedlots, lambs are slaughtered at a set weight or age, to a large extent regardless of sex or breed (Van Der Merwe et al., 2022). Since fat deposition is largely dependent on maturity type (Cloete et al., 2007, 2012) and physiological age (Fitzhugh, 1976; Owens et al., 1993), both of which are influenced by breed (Milne, 2000; Brand et al., 2017, 2018; Van der Merwe et al., 2019), lambs are often slaughtered at a non-optimal point in their production cycle. Finding an accurate way of predicting fat thickness prior to slaughter would therefore benefit producers.

While numerous studies have focused on predicting carcass composition using ultrasound scans of various traits, including fat depth (Fernández et al., 1998; Teixeira et al., 2006; Cadavez et al., 2007; Orman et al., 2008; Thériault et al., 2009; Akdag et al., 2015; Silva, 2017; Dias et al., 2020; Vargas et al., 2021), models to predict fat cover per se are lacking. Van Der Merwe et al. (2022) modelled the fat deposition of various South African sheep breeds and then developed predictive models using age or live weight as inputs, but only focused on pure breeds.

This study aimed to monitor the fat deposition of various crossbred sheep lines using ultrasound and then develop a predictive model using weight or age as inputs to allow lamb producers to accurately determine the optimal slaughter point of their lambs.

## Materials and methods

To obtain the crossbred lambs, a mixed flock of Dohne Merino and Merino ewes on Langgewens Research Farm (33.276 S, 18.705 E) were randomly divided into groups of 20 and the groups mated to rams of various breeds. One group of each maternal line was mated to rams of their own breed, to produce purebred offspring as control groups, while the remaining groups were mated to Dorper, Dormer and Ile de France rams. Eight genotypic groups, Dohne Merino, Dohne x Dorper, Dohne x Dormer, Dohne x Ile de France, Merino, Merino x Dorper, Merino x Dormer and Merino x Ile de France, were therefore included in the trial. Lambing took place in late autumn and early winter, and lambs were weighed and tagged within 24 h of birth. The lambs remained with the ewes in the available pastures until weaning at 100 days of age (34.295 ± 0.561 kg) and had ad libitum access to creep feed from 14 days onwards.

After weaning, four rams and four ewes of each genotype were selected and transported to Elsenburg Research Farm (33.844 S, 18.836 E), where they were placed in individual metabolism cages (1.5 × 2 m) and reared until 1 year of age. Due to spatial constraints, no more than 64 animals could be used in the trial. Upon arriving at Elsenburg, the lambs were adapted to a pelleted feedlot diet (Table 1) over 7 days. After adaptation, the lambs had ad libitum access to feed and potable water throughout the trial.

**Table 1:** Physical and chemical composition, on an as fed basis, of the pelleted feedlot diet lambs received during the trial

Physical composition	Content (g/kg)	*Chemical composition	Content (%)
Maize meal	500.00	Dry matter	88.86
Lucerne hay	361.00	Ash	4.92
Cottonseed oilcake meal	50.00	Protein	16.38
Molasses powder	25.00	Fibre	7.96
Ammonium chloride	5.00	Fat	2.48
Ammonium sulphate	5.00	Acid detergent fibre	9.01
Lime	5.00	Neutral detergent fibre	18.98
Monocalcium phosphate	5.00	Total digestible nutrients	75.71
Common salt	10.00		
Urea	5.00		
Sodium bicarbonate	10.00		
Slaked lime	5.00		
Sulphur	2.00		
Commercial growth promoters and coccidiostat premix	1.20		
Vitamin and mineral premix	1.50		

*Values obtained from proximate analysis of feed (AOAC International, 2002)

Lambs were weighed weekly from 1 month onwards, and scanning of backfat thickness commenced once they reached ~ 80 days of age. No scanning was performed prior to this as the small stature of the lambs and limited fat cover made it difficult to get accurate readings. Scans were performed at the 12–13th rib site on the right side of the sheep as per Thériault et al. (2009) using a Mindray DP-30 V ultrasound scanner with a 7.5 MHz linear transducer probe. Wool was combed from the scanning site, and ultrasound gel was applied to the probe to improve conductivity between the probe and scanning site. These scans continued until 1 year of age, and 39 consecutive readings were obtained for each animal.

From weaning until approximately 220 days, the depth of the right *longissimus thoracis et lumborum* muscle was also measured during the weekly scans. During this period, 16 measurements were made for each animal. After 220 days, the fat and muscle depth had increased to a level that prevented accurate measurements of muscle depth being made.

Statistical analysis of the data was performed using the Statistica 14 software package. The significance level for all analyses was set to 5% (*P* ≤ 0.05).

The fat depth-age and fat depth-live weight datasets were cleaned, and all missing observations or outliers (more than three standard deviations from the mean) were removed. All datasets were normally distributed. The fat depth-age and fat depth-live weight datasets were then fitted to linear, exponential and power equations using the non-linear estimation procedure. Muscle depth was fitted to weight and age, respectively, in the same manner. All assumptions necessary for the application of regression models to the data were met.

After being fitted to the data, the equations were evaluated for goodness of fit using the *R*^2^ and root mean square error (RMSE) statistics to determine the best-fitting equation. The parameters from the equation deemed to best fit the fat depth-age, fat depth-live weight and muscle depth datasets, respectively, were then analysed further using a two-way analysis of variance (ANOVA) test. The ANOVA had sex and genotype set as main effects while also testing for interaction between the main effects. If significant differences existed between groups, a Fisher’s LSD post hoc test was performed to quantify the differences.

## Results

Fat depth-age and fat depth-live weight data were fitted to linear, exponential and power equations, and the goodness of fit of these equations was evaluated.

Evaluating the *R*^2^ values, it was seen that the fitting of the equations to the data was generally satisfactory with values ranging from 0.5 to 0.85. The exponential equation was found to account for the least variation in the data, while the linear fitting of fat depth to age had the highest *R*^2^ values (> 0.77). Specifically looking at equations with weight as an input factor, it was seen that the linear and power equations had similar *R*^2^ and RMSE values, but the linear was found to be marginally better and was therefore selected for further analysis. However, all the equations, with the possible exception of the exponential, appear to be suitable for modelling fat depth.

Since both selected equations were linear, the parameters evaluated were the same for both. The *A* parameter is the intercept value when the input, weight or age, is zero. All the *A* values are negative, and thus only mathematical, and not biological, significance can be attached to them. The *B* parameter, defining the angle of the slope, can however be interpreted biologically, where a higher value would indicate a higher rate of fat deposition and vice versa. The parameter estimates are given as least squares means in Tables 2 and 3.

**Table 2:** Least squares means and standard errors for the parameter values of the linear equation fitted to fat depth and age $\left[FD=A+B\times t\right]$

Main effects		Parameter	
		*A*	*B*
Sex	Ram	− 0.099 ± 0.0187	0.00400 ± 0.0001
	Ewe	− 0.144 ± 0.0187	0.00440 ± 0.0001
	*P*-value	*0.105*	*0.021*
Genotype	Dohne Merino	− 0.067 ± 0.0374	0.00355^d^ ± 0.0002
	Dohne x Dorper	− 0.138 ± 0.0374	0.00468^ac^ ± 0.0002
	Dohne x Dormer	− 0.131 ± 0.0374	0.00449^abc^ ± 0.0002
	Dohne x Ile de France	− 0.106 ± 0.0374	0.00406^bcd^ ± 0.0002
	Merino	− 0.124 ± 0.0374	0.00399^bd^ ± 0.0002
	Merino x Dorper	− 0.149 ± 0.0374	0.00471^a^ ± 0.0002
	Merino x Dormer	− 0.150 ± 0.0374	0.00406^bcd^ ± 0.0002
	Merino x Ile de France	− 0.109 ± 0.0374	0.00418^abc^ ± 0.0002
	*P*-value	*0.793*	*0.007*

Means with different superscripts in the same column (^a–d^) differ significantly (*P* ≤0.05)

*FD* indicates fat depth in cm, and* t* refers to the age of the animal in days

**Table 3:** Least squares means and standard errors for the parameter values of the linear equation fitted to fat depth and live weight $\left[FD=A+B\times W\right]$

Main effects		Parameter	
		*A*	*B*
Sex	Ram	− 0.339 ± 0.0316	0.0167 ± 0.006
	Ewe	− 0.526 ± 0.0316	0.0236 ± 0.006
	*P*-value	< *0.001*	< *0.001*
Genotype	Dohne Merino	− 0.404^ab^ ± 0.0633	0.0187^bc^ ± 0.001
	Dohne x Dorper	− 0.578^bc^ ± 0.0633	0.0218^ab^ ± 0.001
	Dohne x Dormer	− 0.369^a^ ± 0.0633	0.0181^c^ ± 0.001
	Dohne x Ile de France	− 0.336^a^ ± 0.0633	0.0177^c^ ± 0.001
	Merino	− 0.559^bc^ ± 0.0633	0.0231^a^ ± 0.001
	Merino x Dorper	− 0.591^c^ ± 0.0633	0.0240^a^ ± 0.001
	Merino x Dormer	− 0.290^a^ ± 0.0633	0.0188^bc^ ± 0.001
	Merino x Ile de France	− 0.353^a^ ± 0.0633	0.0189^bc^ ± 0.001
	*P*-value	*0.004*	*0.002*

Means with different superscripts in the same column (^a–c^) differ significantly (*P* ≤0.05)

*FD* indicates fat depth in cm, and *W* represents live weight in kilogram

No differences existed between sexes or genotypes for the *A* parameter (Table 2), but differences were present between both sex (*P* = 0.021) and genotype (*P* = 0.007) groups for the *B* parameter in the fat depth-age equation. Ewes (0.00440) had a higher value than rams (0.00400), indicating a greater rate of fat deposition, while Merino x Dorper was the genotype with the highest value (0.00471). It was not significantly higher than Dohne x Dorper, Dohne x Dormer or Merino x Ile de France crosses. Dohne Merino had the lowest *B* parameter value of 0.00355, not significantly lower than the Dohne x Ile de France, Merino and Merino x Dormer groups. No differences were found between the Dohne x Dorper, Dohne x Dormer, Dohne x Ile de France, Merino x Dormer and Merino x Ile de France groups. Similarly, no differences between Dohne x Dormer, Dohne x Ile de France, Merino, Merino x Dormer and Merino x Ile de France were present.

For the fat depth-live weight equation parameters (Table 3), differences were present for both parameters. Rams had a significantly higher *A* parameter value than ewes (− 0.339 vs − 0.526), while genotypes also differed (*P* = 0.004). The Merino x Dormer, Dohne x Ile de France, Merino x Ile de France and Merino x Dormer crosses had the highest values and were not significantly higher than Dohne Merino. Dohne Merino, in turn, did not differ from Dohne x Dorper and Merino. Merino x Dorper had the lowest value (− 0.591), significantly lower than all groups except Dohne x Dorper and Merino.

Ewes had a higher *B* value than rams (0.0236 vs. 0.0167). The highest *B* parameters were found for Merino (0.0231) and Merino x Dorper (0.024), significantly higher than the other groups except for Dohne x Dorper. No differences were present between Dohne Merino, Merino x Dormer and Merino x Ile de France who were not significantly different from the lowest groups, Dohne x Dormer and Dohne x Ile de France.

Linear, exponential and power equations using age and live weight as inputs were once again compared to find the most suitable equation to model loin muscle growth between 100 and 220 days. The power equation with live weight as an input was deemed to provide the best fit, but still did not account for more than approximately 40% of the variation in the data and will therefore not be very accurate in predicting loin muscle depth. Results from the parameter analysis are given in Table 4 below. No significant differences were observed between sexes or genotypes for either parameter.

**Table 4:** Least squares means and standard errors for the parameter values of the power equation fitted to muscle depth and live weight $\left[MD=A\times {W}^{B}\right]$

Main effects		Parameter	
		*A*	*B*
Sex	Ram	0.535 ± 0.072	0.490 ± 0.034
	Ewe	0.396 ± 0.072	0.552 ± 0.034
	*P*-value	*0.175*	*0.207*
Genotype	Dohne Merino	0.404 ± 0.143	0.530 ± 0.068
	Dohne x Dorper	0.401 ± 0.143	0.515 ± 0.068
	Dohne x Dormer	0.341 ± 0.143	0.573 ± 0.068
	Dohne x Ile de France	0.448 ± 0.143	0.584 ± 0.068
	Merino	0.621 ± 0.143	0.503 ± 0.068
	Merino x Dorper	0.527 ± 0.143	0.489 ± 0.068
	Merino x Dormer	0.598 ± 0.143	0.424 ± 0.068
	Merino x Ile de France	0.386 ± 0.143	0.552 ± 0.068
	*P*-value	*0.809*	*0.778*

*MD* indicates muscle depth in cm, and *W* represents live weight in kilogram.

## Discussion

The linear equation was deemed to provide the best fit for both fat depth-age and fat depth-live weight, but the limitations inherent in a linear equation should be borne in mind when interpreting these results. Although the *B* parameter in a linear equation provides a good indication of fat deposition rate, questions can be raised about the biological accuracy. The linearity suggests that fat deposition will take place at a fairly constant rate throughout the animals’ lifetime and that it will never reach an asymptotic value or maximum depth. Since fat deposition is known to accelerate after puberty and again decelerate after maturity is reached (Fitzhugh, 1976), the results are somewhat surprising. It was expected that an exponential or power equation would better fit the data due to the expected post-pubertal acceleration in fat deposition. Previous work on the subject (Van Der Merwe et al., 2022) supported this hypothesis. Instead, the exponential equations provided the least accurate fit of the models tested. This could be due to the limited number of animals in the study, but since Van Der Merwe et al. (2022) also had only four animals per group, it seems unlikely that the difference between the studies is attributable to the number of animals.

Although the number of animals used in the study was limited, two factors have to be borne in mind when interpreting the results. Firstly, the animals were sourced from a resource flock that is genetically linked to various stud flocks. The animals used in the trial can thus be considered representative of the breeds. Secondly, as 39 weekly readings were made per animal, the fat deposition curves of each animal were well-documented. This means that the results obtained in this study are both accurate for the animals included here and can reasonably be extrapolated to the breeds represented by each cross.

It is also possible that this post-pubertal acceleration did not occur because of the high plane of nutrition the lambs were on prior to reaching puberty. Since the lambs were kept in small pens and only exercised outside once a week, their energy requirements would have been lower than that of animals on pasture or in larger pens such as in a feedlot. This in turn would have led to more of the energy from their diets being available to be stored as fat reserves. Fat deposition could therefore have accelerated prior to puberty. On the other end of the age spectrum, it is possible that the expected decline in fat deposition rate near maturity was not realised since the animals had not yet reached maturity. However, this seems unlikely as the modelling of their growth curves (Theron et al., 2023) clearly shows the plateau in growth rate that is associated with reaching maturity.

Regardless, the *B* parameters provide valuable information on the fat deposition of the various groups. Rams generally had a lower fat deposition rate than ewes due to the differences in maturity type. Rams have larger mature weights, thus maturing at a slower rate than ewes (Butterfield, 1988; Owens et al., 1993). Animals that mature later and at a slower rate display lower rates of fat deposition in comparison to early maturing animals (Owens et al., 1993). The same results were obtained by Van Der Merwe et al. (2022).

Both the age- and weight-based models indicated that Merino x Dorper had the highest fat deposition rates, closely followed by Dohne x Dorper. Since the Dorper is a fast-growing, early maturing breed that is known to deposit fat at an early age (Cloete et al., 2007), it is to be expected that crossbreeding with the intermediate maturing breeds Dohne Merino and Merino (Cloete et al., 2012) would lead to the crossbred offspring depositing fat faster than the maternal breeds. Crossbreeding with Dormers, which are indicated as early maturing by Cloete et al. (2012), would be expected to have a similar effect. The age-based model indicates that this would be the case, with both Dohne x Dormer and Merino x Dormer showing greater fat deposition rates than the pure breeds. The weight-based model, however, differs and indicates that both the Dormer crosses would deposit fat at a slower rate than the pure breeds. This model also indicates that the Merino x Dorper cross would deposit fat at a slightly, but not significantly, greater rate than the purebred Merino group. It further indicates that the purebred Merino group will deposit fat faster than the Dohne x Dorper cross. This seems unlikely, given that the Dorper is acknowledged as a breed that deposits fat at a high rate (Milne, 2000; Cloete et al., 2007; Van Der Merwe et al., 2022), while the Merino, being later maturing than the Dorper (Cloete et al., 2007), would be expected to have a lower rate of fat deposition. As the Ile de France is also recognised as an early maturing breed (Snyman, 2014), the same expectations as for the Dormer applied, and again, the age-based model met these expectations with Dohne x Ile de France and Merino x Ile de France depositing fat faster than Dohne Merino and Merino. However, the weight-based model once more differed and indicated that the Ile de France crosses deposited fat at a slower rate than the purebreds.

What can safely be assumed is that biologically accurate predictions of fat depth using age as predictor can be made. Producers with Merino or Dohne Merino flocks who desire to produce lambs with adequate fat cover in a shorter time can consider crossbreeding with Dorpers. It should be borne in mind that this will result in the crossbred offspring producing coarser, coloured wool that is of significantly less value than Merino-type white wool. The fitting of the power curve to loin muscle depth yielded unsatisfactory results, and analysis of the parameter values indicates that no differences exist between sexes or genotypes, which seems unlikely, given that growth rates differ between these groups. It is therefore not advisable to rely on predictions derived from this model.

Given the high statistical accuracy obtained by the fitting of the various equations to the data, it can be seen that fat deposition follows a pattern that can be mathematically predicted and that the equations evaluated in this study can be used to predict fat deposition with a high degree of accuracy. The best-suited model appears to be the linear fitting of fat depth to age, and it should be suitable for practical use, provided the age of the lambs is known. This means that even producers without access to ultrasound technology could use it as a predictive tool to ensure that lambs are slaughtered at an optimal fat cover.

## Data Availability

The data sets generated during the current study are available from the corresponding author upon reasonable request.
